# Supplementary material for: HIV-1 Integration Site Determines the Transcriptional Fate and Persistence of Integrated Proviruses
Source: bioRxiv. 2026 Feb 7:2025.12.26.696579. Originally published 2025 Dec 26. Preprint. [Version 2] doi: 10.64898/2025.12.26.696579 (PMC12776120; doi:10.64898/2025.12.26.696579)
Supplement: 1 [file NIHPP2025.12.26.696579v2-supplement-1.pdf]

Figure S1

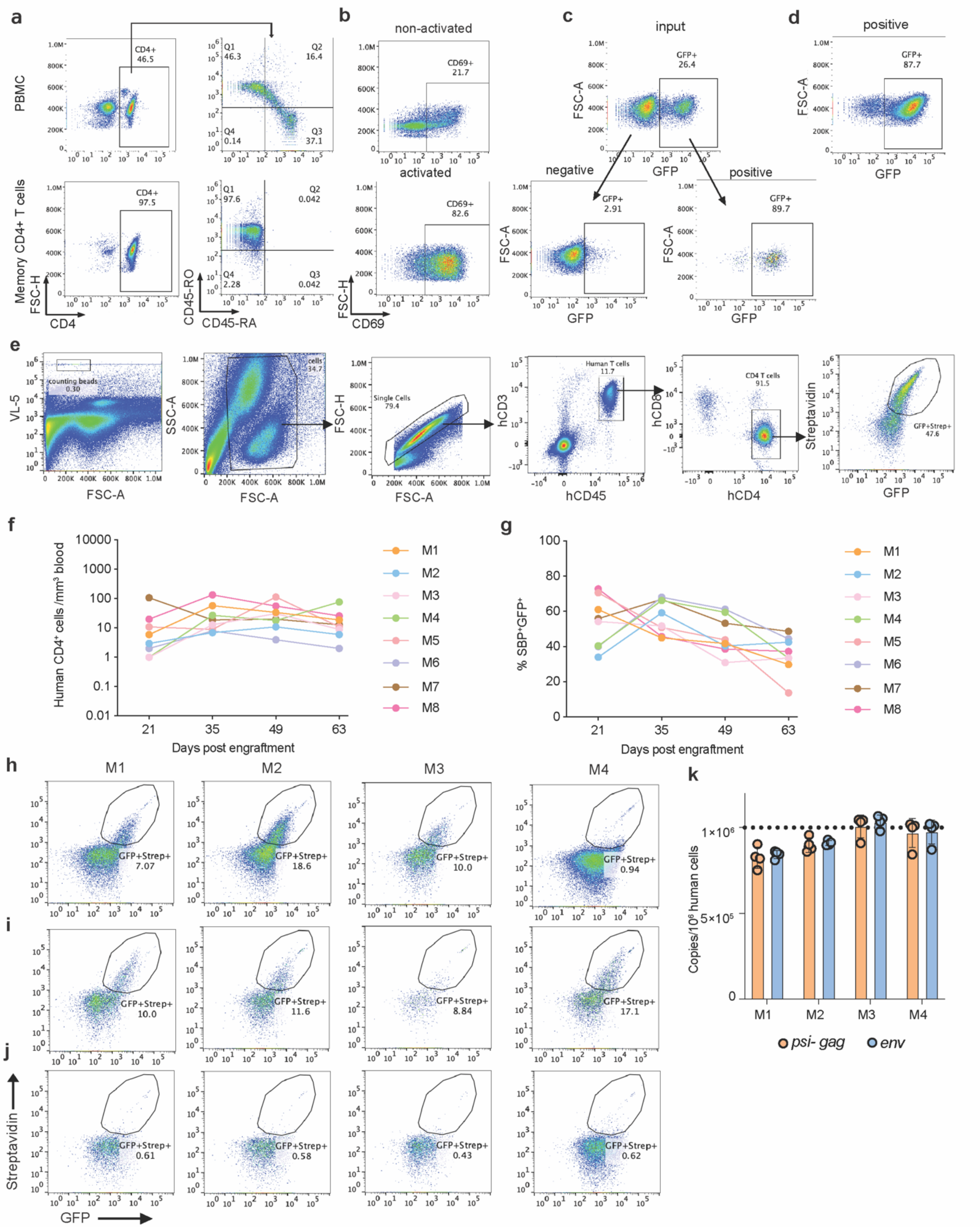

Figure S1. Infection, selection of reporter HIV-1-infected human memory CD4^{+} T cells, and engraftment of NSG mice. **(a)** Flow cytometry plot showing the isolation of human memory CD4^{+} T cells (lower panels)

from peripheral blood mononuclear cells (PBMCs, upper panels) of an HIV-1-uninfected human donor. **(b)** Frequency of activated human memory $CD4^+$ T cells ($CD69^+$) 4 days after activation with anti-CD3/CD28 beads and 2 days after infection with V1/SBP-GFP virus. **(c)** Streptavidin magnetic bead-based cell isolation of V1/SBP-GFP-expressing memory $CD4^+$ T cells. Bead-bound (positive) cells were separated from unbound (negative) cells 48 hours after infection with V1/SBP-GFP virus. **(d)** Percentage of V1/SBP-GFP positive cells 48 hours after sorting, immediately before freezing cells in aliquots for engraftment. **(e)** Flow cytometry gating strategy used for quantification of human $CD4^+$ T cell counts and HIV-1 expression post engraftment. Counting beads for calibration of the quantification were gated based on FSC-A and VL-5 (detector), an Attune NxT detector excited by a 405nm laser with an emission filter at 710/50nm. The figure reports the percentage of transcriptionally active HIV-1-infected (SBP^+GFP^+) cells in the $hCD3^+/hCD45^+/hCD4^+$ human cell compartment in a typical mouse peripheral blood sample. **(f, g)** Quantification of the numbers of human cells over ~2 months following mouse engraftment; total number of human cells (**f**) and percentage of transcriptionally active HIV-1 infected (SBP^+GFP^+) human cells (**g**) over time in multiple animals. **(h-j)** Percentage of transcriptionally active HIV-1 infected (SBP^+GFP^+) human cells (gated on $hCD3^+/hCD45^+/hCD4^+$ cells) in mouse spleen (**h**), lung (**i**), and bone marrow (**j**) of individual animals harvested 2 months after engraftment. **(k)** Quantification of *psi-gag* and *env* DNA copies per million human cells in spleen from each engrafted mouse.

Figure S2

a

Sample	Sample processing		Viral-host junction	Total integration sites	Unique integration sites	% clonal
Pre-infusion	unsorted		5' LTR	3789	5239	3.10%
			3' LTR	3887		
M1			5' LTR	1338	117	54.70%
			3' LTR	1258		
M2			5' LTR	1928	183	63.90%
			3' LTR	2066		
M3			5' LTR	1713	190	50%
			3' LTR	1797		
M4			5' LTR	1166	158	44.30%
			3' LTR	1087		
M5	sorted	Active	5' LTR	2191	328	23.17%
			3' LTR	2594		
		Latent	5' LTR	230	133	25.56%
			3' LTR	362		
M6		Active	5' LTR	971	85	35.30%
			3' LTR	1110		
		Latent	5' LTR	1022	46	41.30%
			3' LTR	1514		
M7		Active	5' LTR	971	235	28.51%
			3' LTR	1110		
		Latent	5' LTR	230	179	19.55%
			3' LTR	362		
M8		Active	5' LTR	2191	841	8.44%
			3' LTR	2594		
		Latent	5' LTR	1022	657	12.48%
			3' LTR	1514		

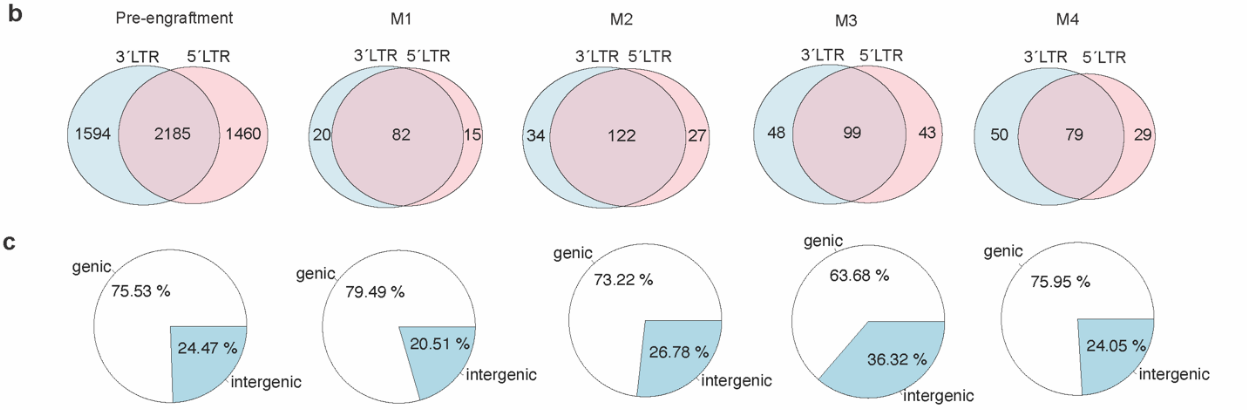

Figure S2. Identification of proviral integration sites in HIV-1 infected human memory $CD4^+$ T cell populations pre- and post-engraftment. **(a)** Table showing the number of unique integration sites retrieved from human cells harvested from the spleen of individual, engrafted mice. Cells recovered from mouse spleens for animals M1-M4 were not sorted, while human cells recovered from mice M5-M8 were sorted based on SBP/GFP expression. The 'percent clonal' column reports the proportion of the total number of unique integration sites found within a sample that were detected in more than one well out of the 96 wells tested. **(b)** Comparison and overlap in the numbers of integration sites retrieved by either 5´LTR or 3´LTR viral-host junction amplification strategies for pre- and post-engraftment cell populations. **(c)** Distribution of integrated proviruses in genes and intergenic regions in pre- engraftment cells and post-engraftment cells from individual mice.

Figure S3

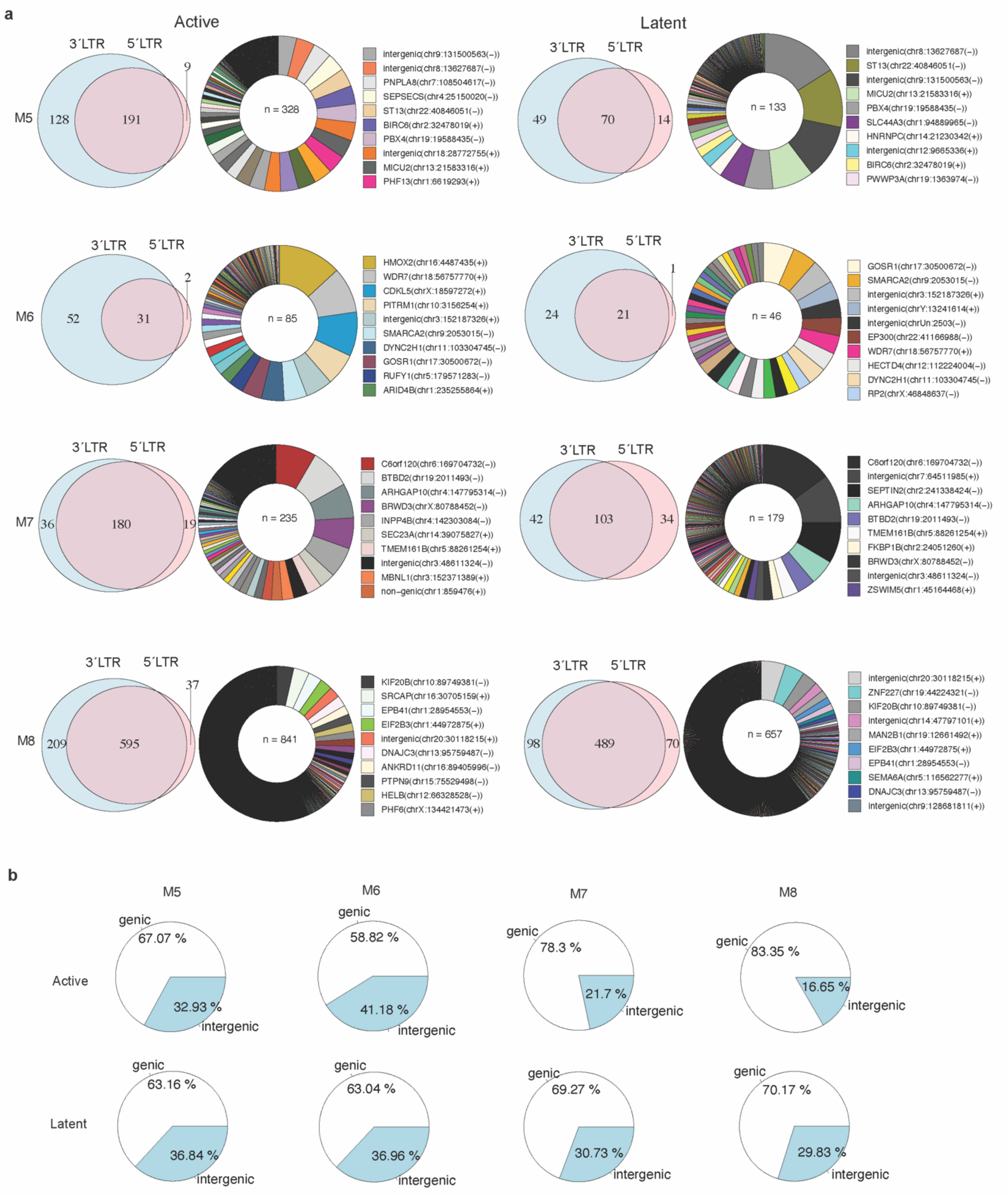

Figure S3. Integration sites identified in HIV-1 infected human memory CD4^{+} T-cells post-engraftment with active and latent proviruses. **(a)** Comparison of integration sites retrieved by 5´LTR and 3´LTR viral-host junction amplification. Circos plots showing the relative proportion of expansion of each clone in post-engraftment human cells from individual mouse spleens, sorted into populations with active and latent

proviruses. Each clone is color-coded according to its proviral integration site. Each integration site is labelled as the name of the gene with integrated provirus or as intergenic if integration was outside of the gene, followed by the chromosome name, the genomic coordinate, and the host DNA strand (positive or negative). **(b)** Distribution of integrated proviruses in genes and intergenic regions in pre- and post-engraftment samples from individual mice.

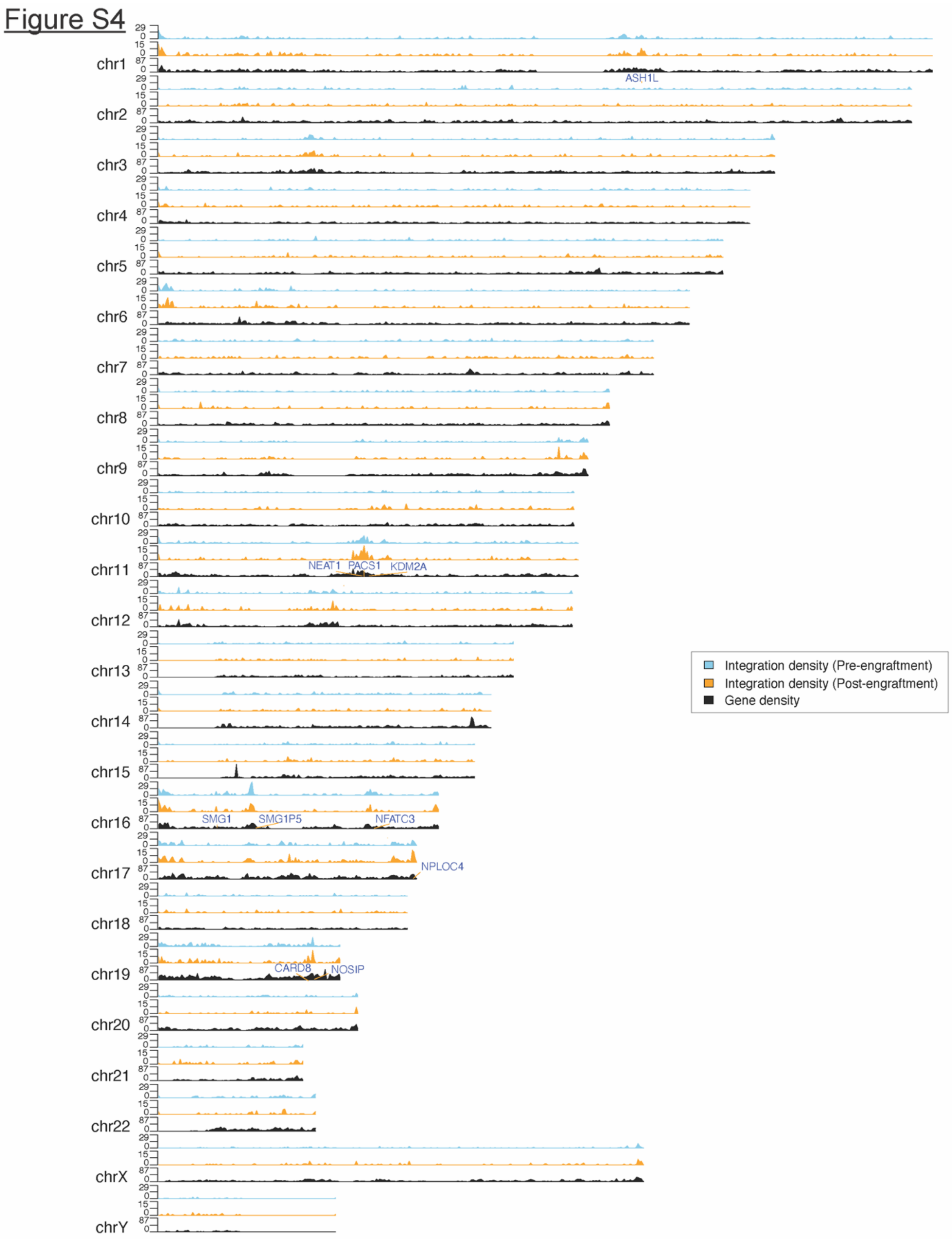

Figure S4. Gene density and integration frequency for human chromosomes and integration sites found in the pre- and post-engraftment cell populations. Genes with >5 unique integration sites post-engraftment are highlighted. The stacked y-axes represent the number of genes (0-87), the number of integrations in pre-(0-29), and post- (0-15) engraftment cell populations.

Figure S5

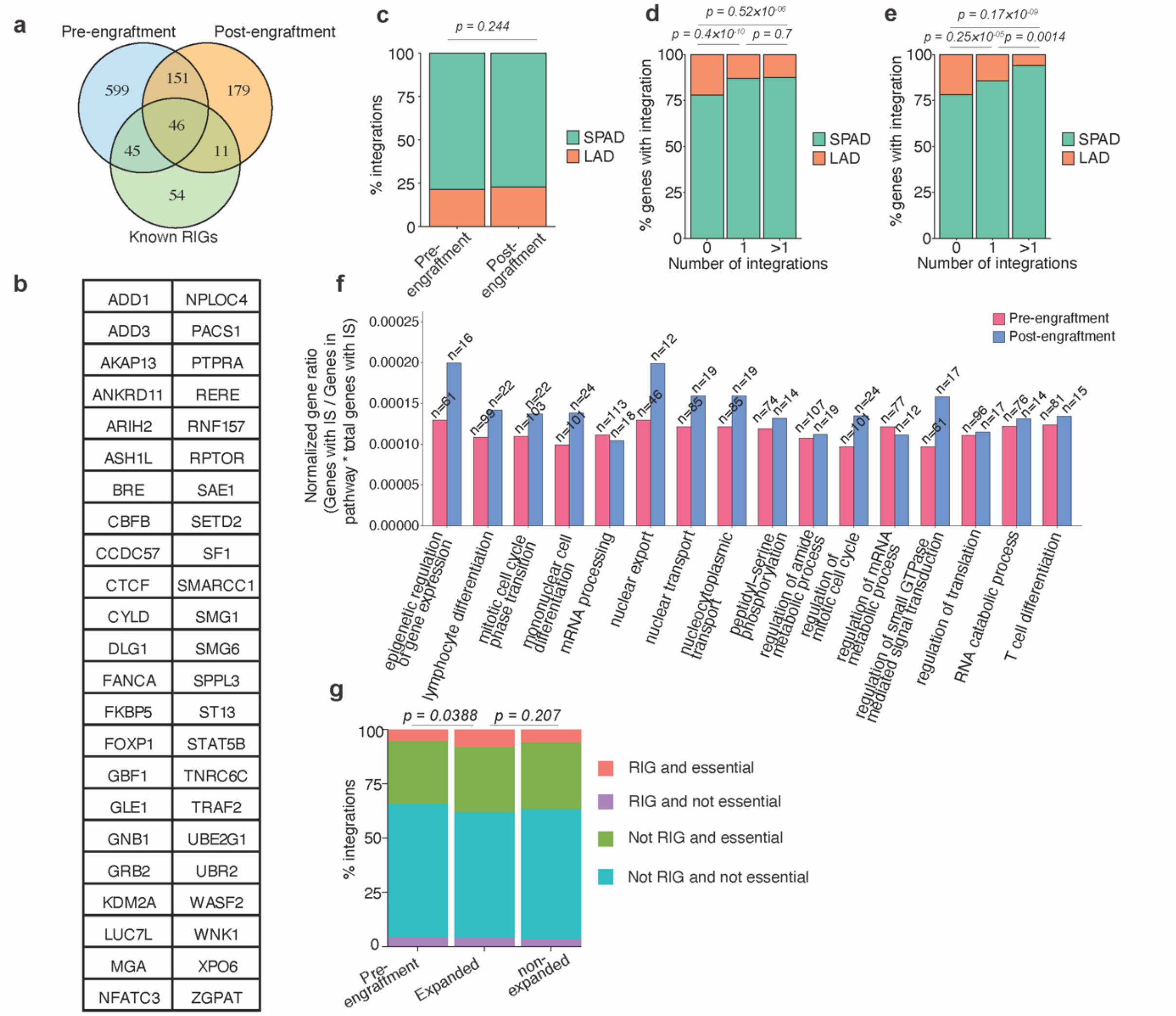

Figure S5. (a) Overlap of genes with >1 integration identified in the pre- and post- engraftment cell populations with genes designated RIGs in previous studies[51,55,56]. **(b)** List of 46 genes that overlap between the pre- /post-engraftment and the previously known RIGs. **(c)** Percentage of proviral integration in SPAD and LAD for integration sites obtained from pre- and post-engraftment populations. **(d, e)** Distribution in SPAD and LAD genomic regions for genes with 0, 1 or >1 integrations in pre- (d) and post-engraftment (e) samples. **(f)** Comparison of genes with HIV-1 integration sites in pre- and post-engraftment samples for association with various pathways as defined by gene ontology (GO) enrichment analysis. The top 15 enriched pathways based on the post-engraftment genic integration sites dataset were selected, and their respective enrichment in both pre- and post-engraftment samples compared. **(g)** Genic integration site distribution in genes that are RIG and essential, RIG and not essential, not RIG and essential, and not RIG and not essential, for pre-engraftment, as well as expanded, and non-expanded clones in post-engraftment datasets. P values determined by Pearson's Chi-squared test.

Figure S6

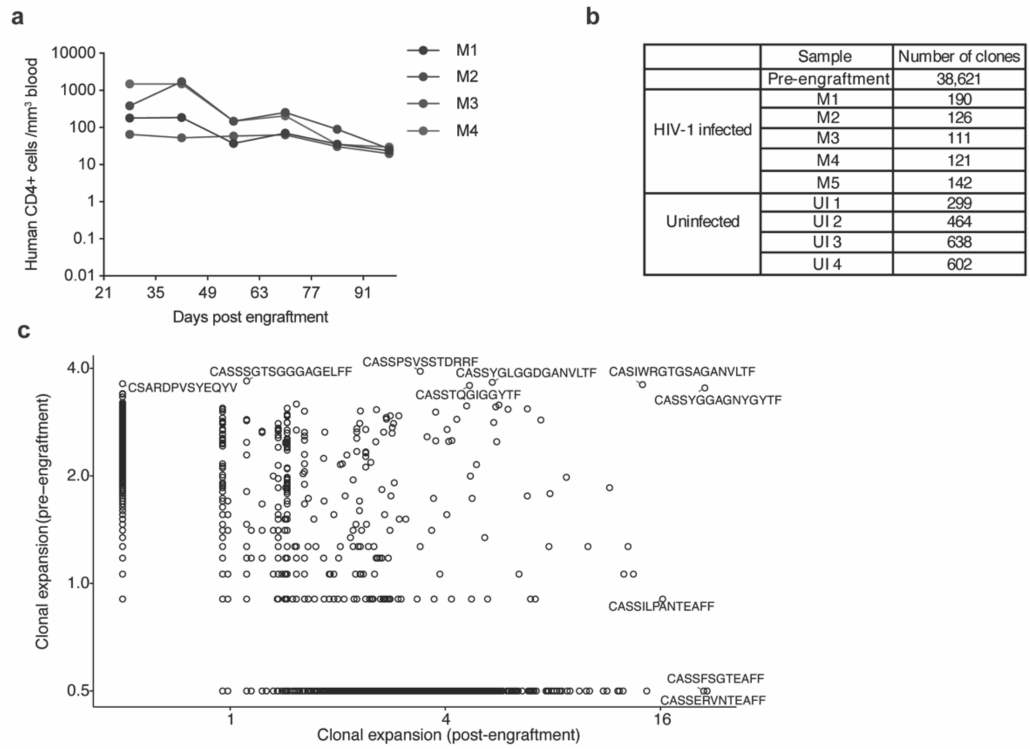

	Sample	Number of clones
	Pre-engraftment	38,621
HIV-1 infected	M1	190
	M2	126
	M3	111
	M4	121
	M5	142
Uninfected	UI 1	299
	UI 2	464
	UI 3	638
	UI 4	602

Figure S6. (a) Quantification of the number of human cells following engraftment of uninfected human memory $CD4^{+}$ T cells over time in multiple animals. Each mouse was sacrificed at 98 days to measure the clonal expansion of individual T cell clones. **(b)** Number of T cell clones retrieved from pre-engraftment, HIV-1-infected, and uninfected post engraftment cell populations from individual mouse spleen samples. **(c)** Comparison of TCR sequence abundance in pre- and post-engraftment cell populations. The dot plot shows the total expansion of all clones retrieved from the uninfected and infected grafts. Each dot represents a T cell clone marked by its CDR3 TCR sequence. TCR sequence not detected in one of the two populations are assigned an arbitrary value of 0.5 for representation on a logarithmic scale.

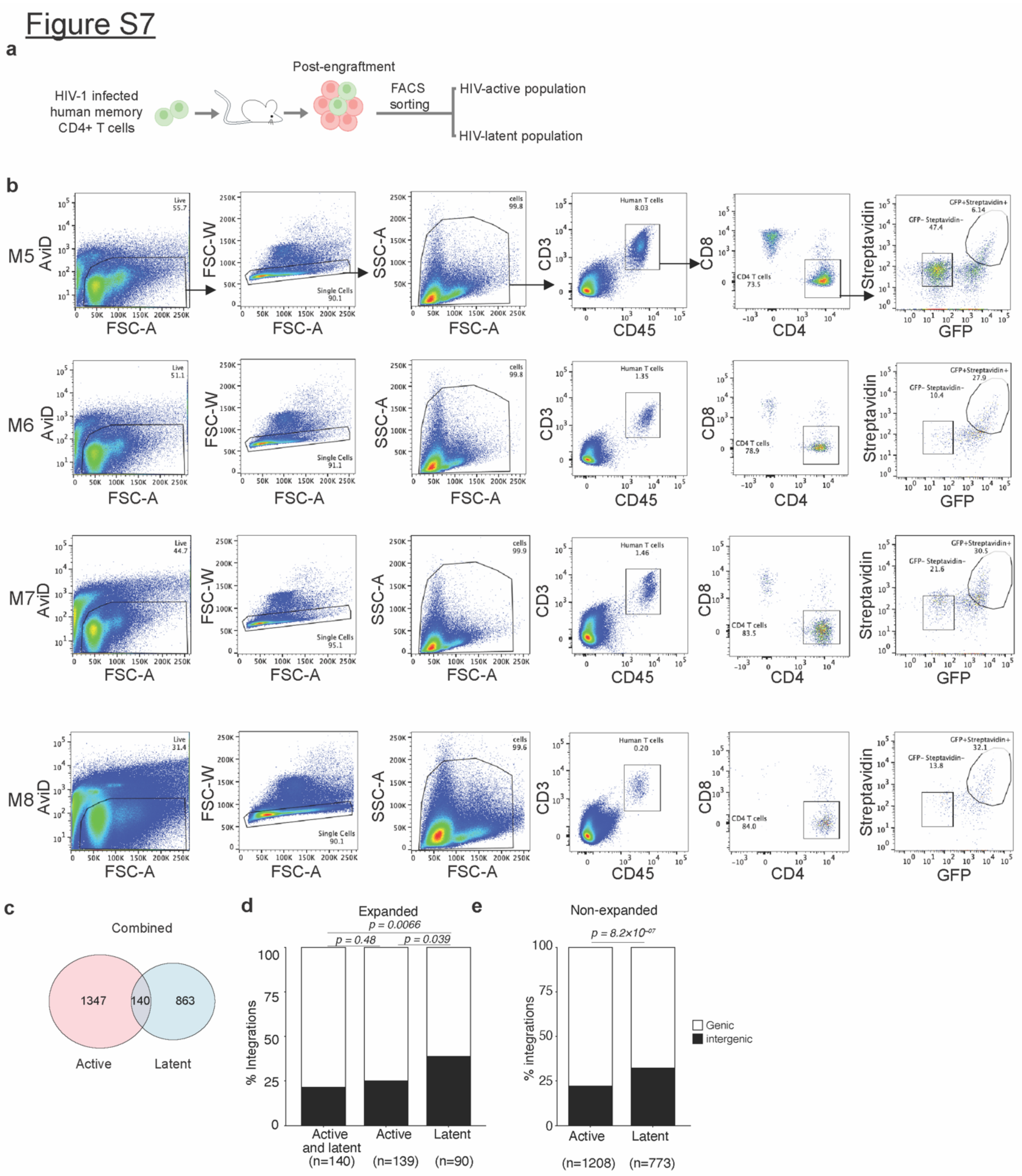

Figure S7. (a) Procedure for sorting of cell populations with active and latent HIV-1 proviruses post-engraftment. **(b)** Flow cytometry gating strategy to sort active ($GFP^{+}SBP+$) and latent ($GFP^{-}SBP^{-}$) HIV-1-infected cell subpopulations post-engraftment. **(c)** Overlap analysis of integration sites found in sorted active and latent cell populations post-engraftment, pooled data from four mice. **(d, e)** Genic and intergenic distribution of integration sites obtained in active, latent, or active and latent proviruses for expanded and non-expanded cell clones, respectively. P-value denotes Fisher's exact test.

Figure S8

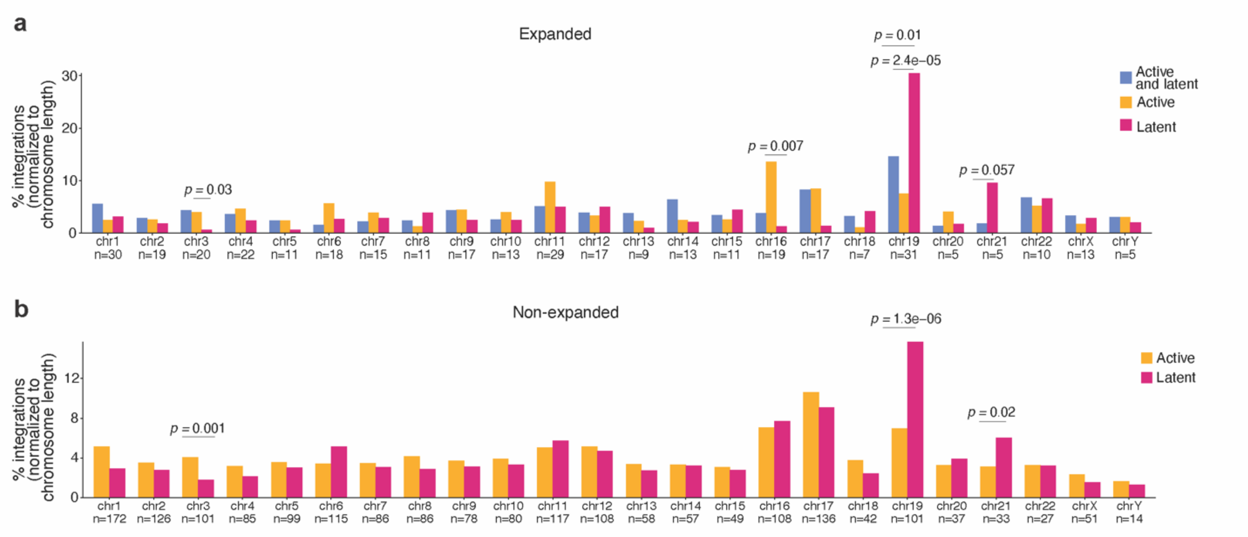

Figure S8. (a and **b)** Distribution of active, latent, or active and latent provirus integration sites found in expanded and non-expanded cell clones on human chromosomes. P-value denotes Fisher's exact test.

Figure S9

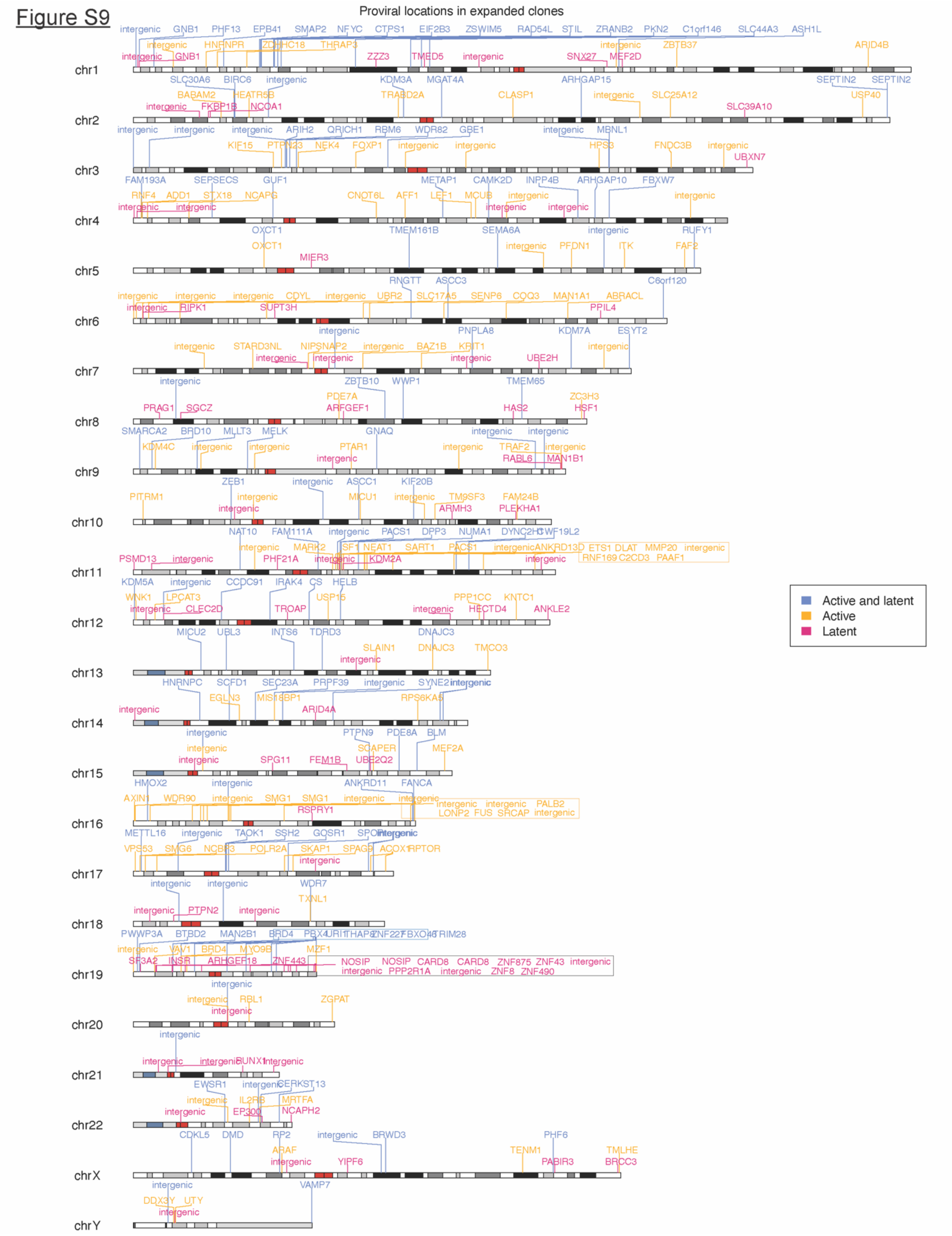

Figure S9. Karyoplot showing the chromosomal distribution of active, latent, or both active and latent provirus integration sites identified in expanded clones. Each ideogram represents a chromosome, and

integration sites are mapped to their genomic coordinates. Proviruses are colored according to transcriptional status. Cytogenetic bands are shown based on Giemsa staining patterns following UCSC Genome Browser and ISCN conventions. Cytoband color key shows white (gneg), gene-rich GC-rich euchromatin; light to dark gray (gpos25–gpos100), increasing Giemsa-positive intensity indicating progressively AT-rich, gene-poor heterochromatin; red, centromeric regions; blue, secondary constrictions containing rRNA gene clusters; gray (gvar), variable heterochromatin.

Figure S10

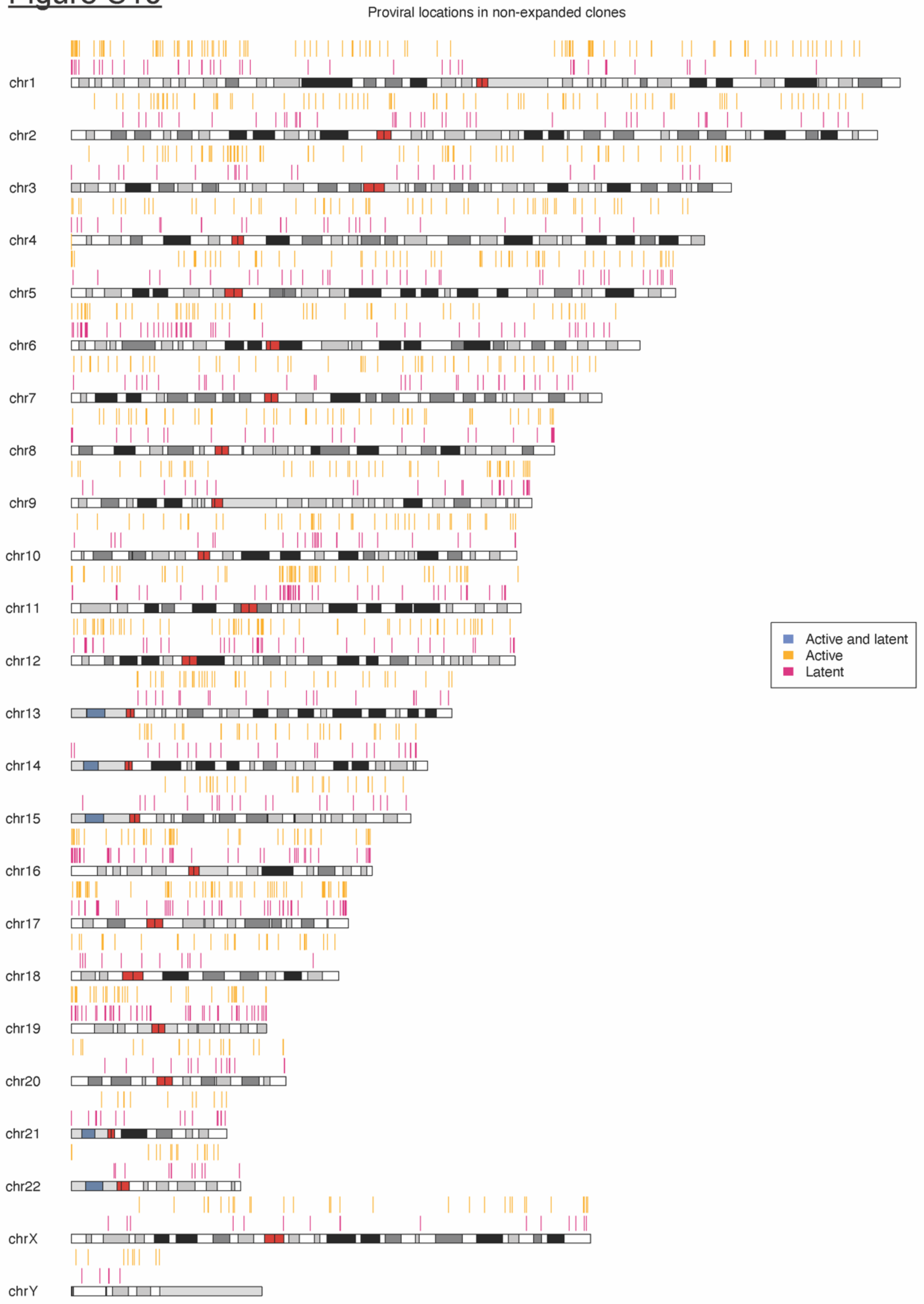

Figure S10. Karyoplot showing the chromosomal distribution of active and latent provirus integration sites identified in non-expanded clones. Each ideogram represents a chromosome, and integration sites are mapped to their genomic coordinates. Proviruses are colored according to transcriptional status, and

multiple integrations within non-expanded clones are displayed on the same chromosome. Cytogenetic bands are shown based on Giemsa staining patterns as described for Figure S9.

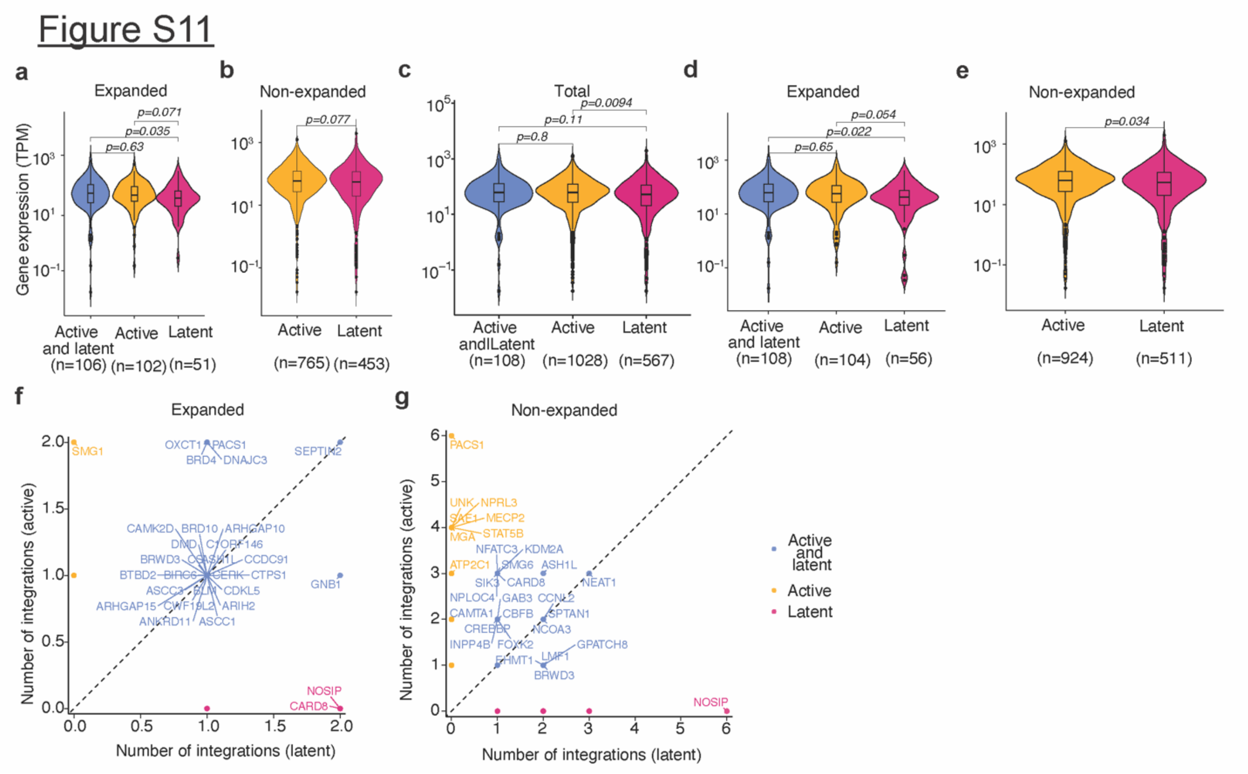

Figure S11. (a and b) Expression levels of genes with active, latent, or active and latent proviruses for expanded and non-expanded cell clones. **(c-e)** Gene expression associated with individual active, latent, or active and latent proviruses for expanded, non-expanded, and total cell clones. P value denotes the Wilcoxon rank-sum test with Bonferroni correction for multiple comparisons. **(f** and **g)** Number of distinct integrations in genes with active and latent proviruses found in expanded and non-expanded cell clones.

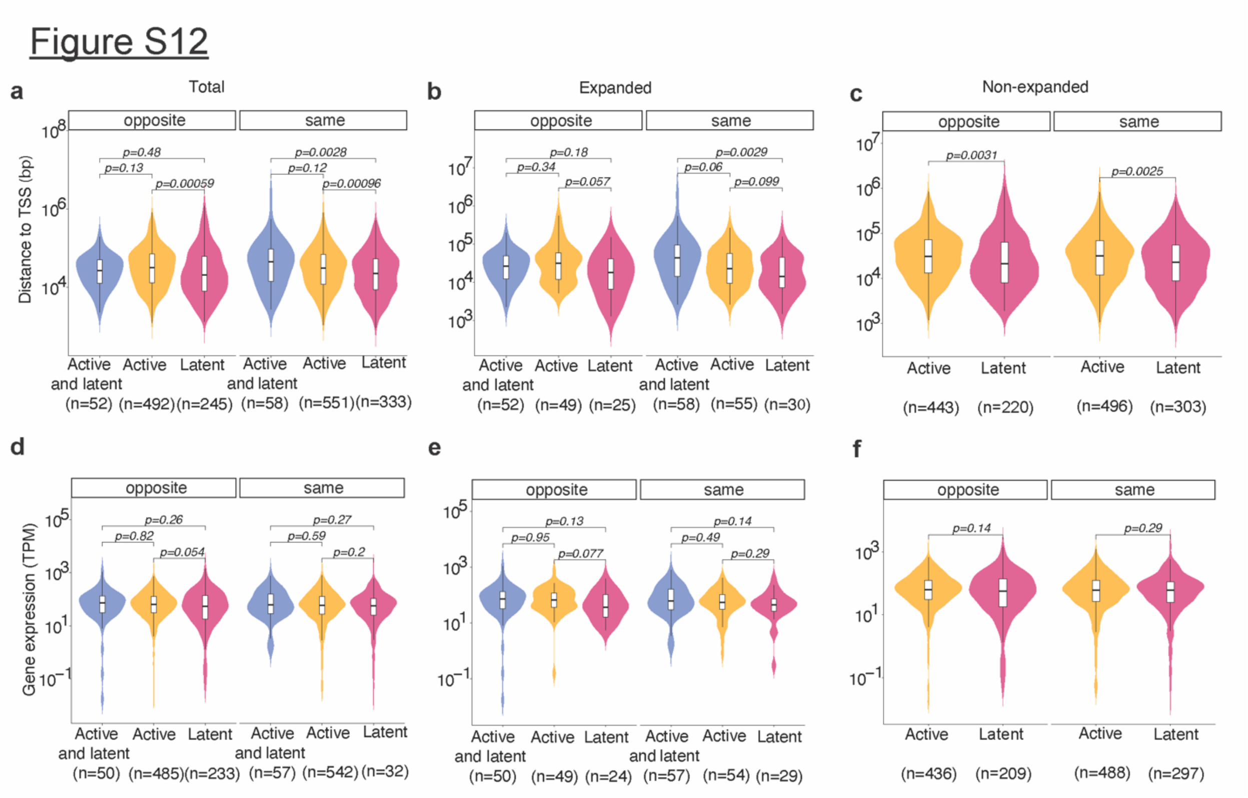

Figure S12. (a-c) Distance of integrated genic proviruses from the TSS for proviruses integrated in the same or opposite orientation of the TSS in the expanded, non-expanded, and total cell clones. **(d-f)** Expression of genes with integrated proviruses in the same or opposite orientation with respect to host transcription in expanded, non-expanded, and total cell clones. P value denotes the Wilcoxon rank-sum test with Bonferroni correction for multiple comparisons.

Figure S13

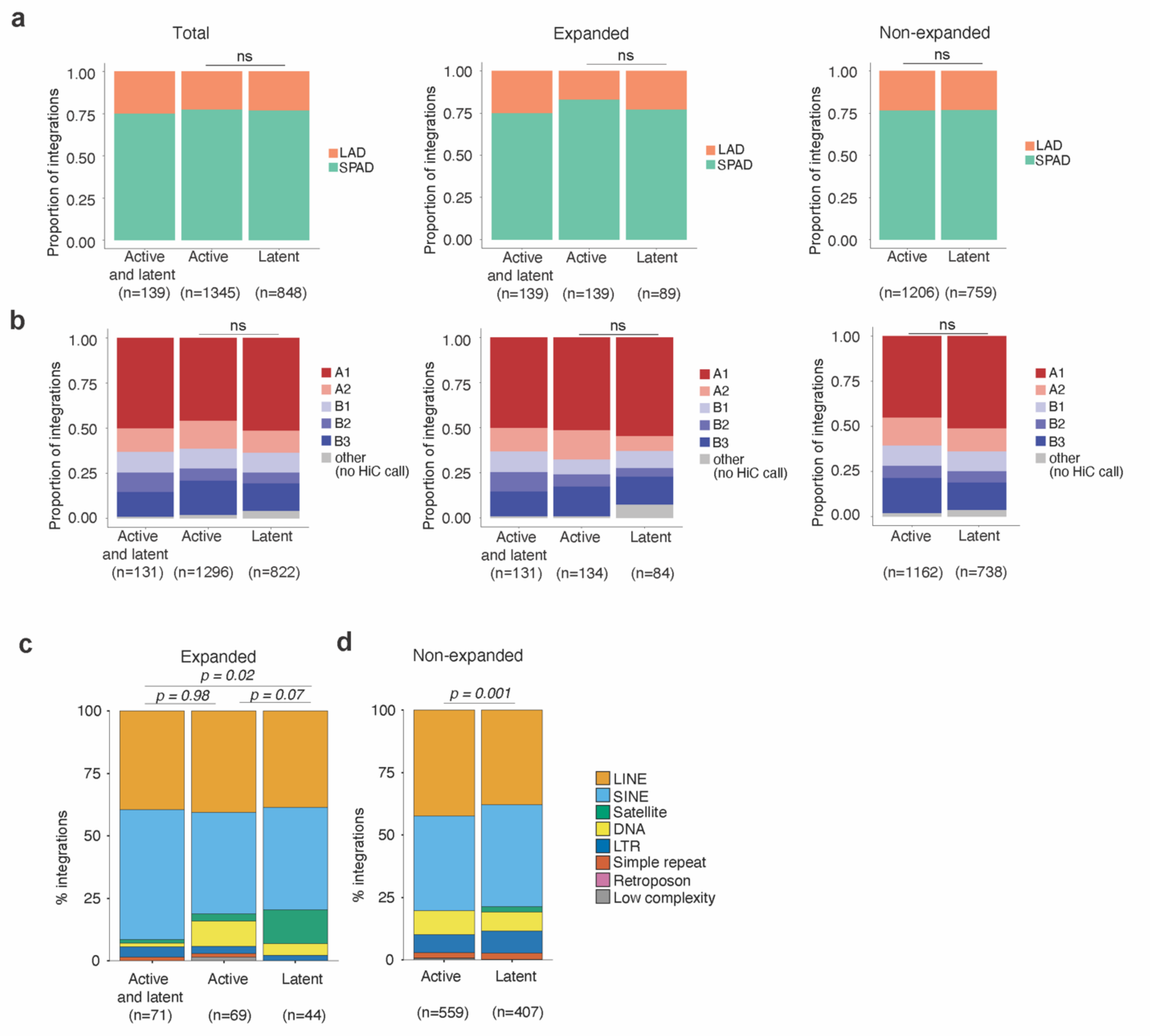

Figure S13. (a) Proportion of SPAD and LAD annotation for associated with active, latent, or active and latent proviruses in total, expanded, and non-expanded cell clones. **(b)** Proportion of active, latent, or active and latent proviruses found within chromatin structural compartments A and B and their respective sub-compartments as determined by Hi-C sequencing data for expanded, non-expanded, and total cell clones. P-value denotes the two-proportion z-test. **(c** and **d)** Percent of proviruses in distinct chromosomal repeat regions in the human genome for active, latent, or both active and latent proviruses in expanded and non-expanded cell clones. P-value denotes the two proportions z-test for the satellite repeat elements class.

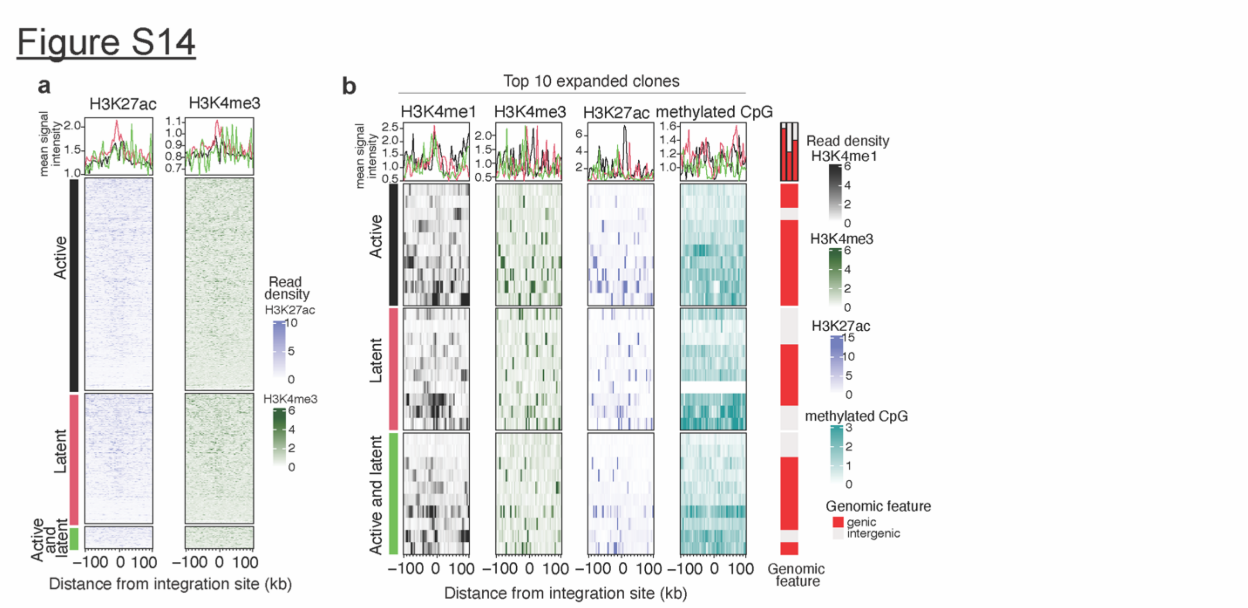

Figure S14. (a) Heat map showing levels of histone marks found up to 100kb upstream and downstream of the integration sites of active, latent, or both (active and latent) proviruses. The line plot above the heat map shows the mean signal across all integration sites found within the active (black), latent (pink), and both (green) categories. The heatmaps for each group are arranged from top to bottom according to the highest to lowest mean signal intensity for H3K36me3, as in Fig 5a. **(b)** Heat map showing levels of histone marks found up to 100kb upstream and downstream of the integration sites of active, latent, or both (active and latent) proviruses for the top 10 expanded clones in each category.

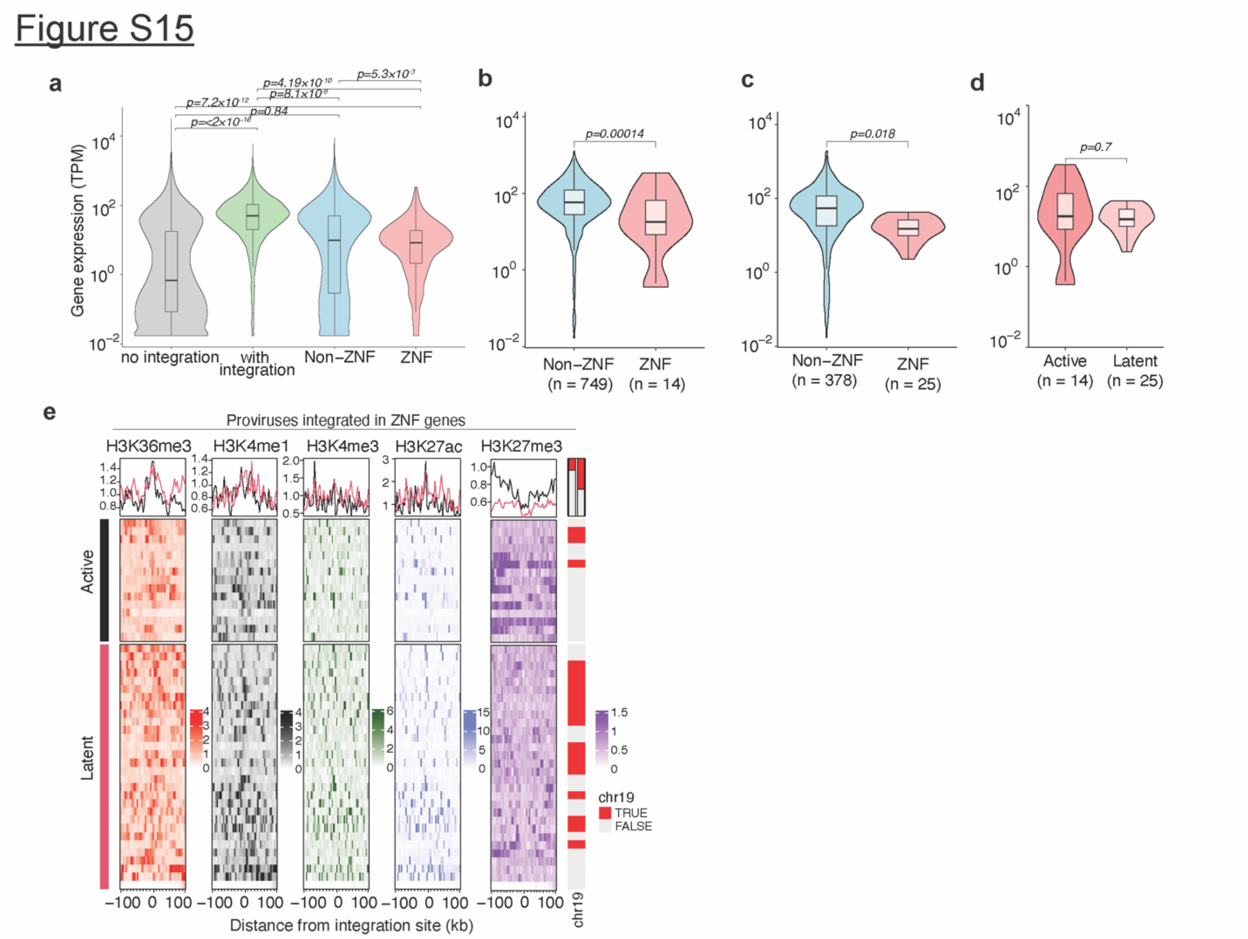

Figure S15. (a) Comparison of the level of expression of genes with or without HIV-1 integration, non-ZNF, and ZNF genes. **(b** and **c)** The expression of ZNF genes as compared to non-ZNF genes with either the active (**b**) or latent (**c**) proviruses. **(d)** Comparison of the expression of ZNF genes with active or latent proviruses. The box inside the violin plot shows median, interquartile ranges, and minimum/maximum values. P values were determined by the pairwise Wilcoxon rank-sum test. **(e)** Heat map showing levels of histone marks found up to 100kb upstream and downstream of the integration sites of active, latent, or both (active and latent) proviruses for proviruses integrated in ZNF genes.
